# Novel Biological Strategies for Melanoma Therapy: A Focus on lncRNAs and Their Targeting

**DOI:** 10.3390/cancers17081273

**Published:** 2025-04-09

**Authors:** Francesca Maria Orlandella, Rosaria Arcone, Neila Luciano, Giuliana Salvatore, Maria Letizia Motti

**Affiliations:** 1 Department of Medical, Human Movement and Well-Being Sciences, University of Naples Parthenope, 80133 Naples, Italy; francescamaria.orlandella@uniparthenope.it (F.M.O.); rosaria.arcone@uniparthenope.it (R.A.); neilaluciano14@gmail.com (N.L.); 2 CEINGE-Biotecnologie Avanzate “Franco Salvatore”, 80131 Naples, Italy

**Keywords:** melanoma, lncRNAs, phytochemicals, RNA-based therapy, nanotechnologies

## Abstract

Increasing evidence revealed that restoring the correct expression of lncRNAs could have implications in the management of melanoma patients. In this context, here, we aim to dissect the main characteristics of lncRNAs altered in melanoma and their crosstalk with the signaling pathways involved in the progression of this disease. We also highlight the role of nucleic acid-based techniques and natural compounds (i.e., phytochemicals) as a therapeutic tool to increase or silence their expression in cancer cells. Finally, we explore the advances in nanotechnologies as delivery systems to efficiently carry these chemicals into cancer cells, thus limiting their potential off-target effects. The analysis of the literature showed that HOTAIR, MALAT1, and H19 are the oncogenic lncRNAs most studied in melanoma, while MEG3 is an important tumor suppressor decreased in this cancer. The aberrant expression of these lncRNAs affects several hallmarks of cancer, e.g., proliferation, motility, and epithelial to mesenchymal transition, promoting the melanoma plasticity and drug resistance. In this frame, siRNA, antisense oligonucleotide, and CRISPR-Cas9 genome editing appear to be the most effective nucleic acid strategies to restore the physiologic expression of lncRNA, while curcumin, resveratrol, and quercetin are the main phytochemicals able to target and influence the expression of lncRNAs altered in cancer. Overall, this study provides a comprehensive overview regarding the role of lncRNAs in the phenotype plasticity of melanoma cells and their potential targeting using RNA-based therapy and natural products.

## Simple Summary

Although available treatments for metastatic melanoma have significantly improved the clinical outcome of patients, there is still a portion of patients whose tumor progresses. Therefore, new therapeutic approaches for melanoma are needed, which could include targeting specific lncRNAs altered in this neoplasm. Indeed, the lncRNAs aberrant expression influences several hallmarks of melanoma cells and contributes to the acquisition of drug resistance. This review focuses on the main lncRNAs altered in melanoma, highlighting the consequences of their aberrant expression on the phenotypic plasticity of melanoma cells. Furthermore, we investigated their possible targeting using nucleic acid-based techniques or natural products such as phytochemicals. Finally, we explored the advances in nanotechnology for the specific delivery of lncRNAs into cancer tissues and to avoid potential side effects.

## 1. Introduction

Malignant melanoma is a highly aggressive tumor with a great capacity to metastasize. The main risk factor for its development is prolonged exposure to ultraviolet (UV) radiation [1]. In recent years, the incidence of patients with a melanoma diagnosis has reached approximately 5% of all cancer cases, not only for the increased exposure to UV radiation but also for increased surveillance [2].

Most melanomas are sporadic and occur due to somatic genetic alterations mainly but not only caused by sun exposure. The most frequent mutations are in the *BRAF* gene, commonly the V600E in exon 15, present in almost 50% of all patients with cutaneous melanoma. There are also less frequent mutations in V600 codon such as V600K, V600R, and V600D [2,3]. Additionally, approximately 25% of all cutaneous melanoma patients harbor the *NRAS* mutation [4]. The *BRAF* and *NRAS* mutations are mutually exclusive [4] and result in hyperactivation of mitogen-activated protein kinase (MAPK) signaling, promoting cell proliferation and survival that lead to tumor progression [4,5,6].

The melanomagenesis is determined not only by mutations in oncogenes and tumor suppressors, but also by epigenetic mechanisms such as acetylation and methylation of histones determining chromatin remodeling, methylation in gene promoters, deregulation of non-coding RNAs [7], including microRNAs (miRNAs) and long non-coding RNA (lncRNA) [8,9,10].

The tumor microenvironment (TME) can also strongly influence the behavior of melanoma cells, regulating tumor progression, response to therapies, and the development of resistance mechanisms [11]. Moreover, melanoma progression is based on the ability of melanoma cells to change phenotype by the dynamic activation of potentially reversible microenvironment-driven molecular programs [12]. The existence of a high degree of phenotypic plasticity is a distinctive feature of malignant melanoma. The interaction with TME together with the reaction to antitumor therapies are able to strongly influence tumor development by determining a “phenotypic switching” of melanoma cells, correlated to their extreme plasticity [12].

### Melanoma Therapy

To date, surgical treatment is the best intervention for primary melanoma. However, colonization and growth of tumor cells in secondary sites still represent the main cause of mortality also due to the development of resistance to the pharmacological treatments in use [13].

The identification of melanoma genetic mutations has been very important for the development of new targeted therapies offering a valid treatment for late-stage patients. In clinical practice, inhibitors that target components of the MAPK pathway are commonly used, in particular, BRAF inhibitors (encorafenib, dabrafenib, vemurafenib) and MEK inhibitors (trametinib, binimetinib, cobimetinib), individually or in combination [14,15]. However, patients who respond well to these therapies almost always develop acquired resistance to these drugs after a few months, limiting their long-term efficacy [16]. Usually, these resistance mechanisms are determined by acquiring the ability to escape the inhibition of the pathway through the onset of new mutations or epigenetic modifications [9,13,17,18]. For this reason, the identification of new molecular targets that could bypass the developed resistance mechanisms has become a primary need [19,20,21].

Over the last decade, melanoma therapy has made significant progress owing to the advent of immunotherapy that is mainly based on the use of immune checkpoint inhibitors (ICIs) [22]. Since 2011, when they were first approved by the Food and Drug Administration (FDA), ICIs have been routinely used as antitumor therapy. These monoclonal antibodies work by blocking specific immune checkpoints, including PD-1 (pembrolizumab, nivolumab), PDL-1 (atezolizumab), CTLA-4 (ipilimumab), and lymphocyte activation gene 3 protein, LAG-3 (relatlimab). These antibodies, alone or in combination, have demonstrated high efficacy in clinical trials for advanced melanoma [23,24,25].

Checkpoint inhibitor immunotherapy has completely changed the landscape of melanoma therapy. However, not all patients with metastatic melanoma respond in the same way. Some remain insensitive to immunotherapy, showing primary resistance; other patients become resistant after a period of effective response to therapy, thus developing secondary or acquired resistance [26]. It is likely that a group of tumor cells with genetic and epigenetic traits that allow escape from the immune system, when subjected to selection by therapy, may determine the development of resistance [27]. Currently, the mechanisms that determine acquired resistance in melanoma patients are still not completely known. The use of combinations of different therapies could be more effective in overcoming the emergence of resistance, although there may be variability among patients [28]. Within this frame, the aberrant expression of lncRNAs regulating several hallmarks of cancer, promoting the phenotypic plasticity of melanoma cells that contributes to the acquisition of drug resistance, suggest that these molecules could represent a novel potential target for melanoma therapy.

In light of this, the aim of this review is to dissect recent findings regarding the lncRNAs deregulated in melanoma, identifying their role in the biological mechanisms of melanoma plasticity, in tumor progression, and in the acquisition of drug resistance. Importantly, we also explore their specific targeting by the delivery of chemical acid nucleic molecules or by the administration of natural compounds.

## 2. Long Non-Coding RNA in Melanoma

Included in the non-coding RNA family, the long non-coding RNAs (lncRNAs) are molecules longer than 200 nucleotides that are able to regulate gene expression at epigenetic, transcriptional, and translational level [29].

Thus, coherently to the pivotal role of lncRNAs in the balance of cell biology, their aberrant expression has been described in cancer patients where they are implicated in the insurgence and progression of this disease [30,31,32,33].

Indeed, through their interaction with chromatin structure, RNA, and protein, these molecules regulate several phenotypes of cancer cells such as proliferation, apoptosis, migration and invasion, plasticity, EMT, and stemness [30,32,34].

As consequence, various studies suggest the use of lncRNAs as a promising novel strategy against several types of cancer, including melanoma [35,36,37,38,39].

### 2.1. Role of lncRNAs on Melanoma Phenotype

The aberrant expression of lncRNAs in melanoma tissues influences mainly cell proliferation and invasion that exploit different mechanisms of action, as summarized in Table 1.

For instance, several studies revealed that the majority of lncRNAs act as competing endogenous RNAs (ceRNAs), sponging a specific miRNA that hinders the binding with the mRNA target [68,69].

In this context, the oncogenic lncRNAs BANCR [40], SNHG5 [41], ATB [42], and LINC0062 [43] influence the expression of NOTCH-2, TRPC3, YAP1, and ELK3 by sponging specific miRNAs (i.e., miR-204, miR-26a-5p, miR-590-5p, and miR-890, respectively), modulating the proliferation, motility, and tumor growth of melanoma cells.

PVT1 also exerts an oncogenic role in melanoma cells by sponging and affecting the action of the tumor suppressor miR-26b [62].

NEAT1 is a well-described lncRNA in melanoma that exerts its oncogenic activity by regulating the expression of E2FR3, KLF3, and SMAD2 by directly binding miR-495-3p [44], miR-23a-3p [45], and miR-200-3p [46].

Also, the lncRNA FOXD3-AS1 acts as a ceRNA in melanoma, since by sponging miR-127-3p [47] and miR-325 [48] it promotes cell proliferation and motility by increasing the expression of FJX1 and of MAP3K2, respectively.

Likewise, in malignant melanoma, the sponging of the lncRNA TUG1 with miR-129-5p [49], miR-29c-3p [50], or miR-145-5p [51] increased the expression of AEG-1, RGS1, and SOX2, respectively enhancing proliferation, tumor growth, and metastasis formation.

By sponging miR-499-5p [52] and miR-208 [53], MEG3 is a tumor-suppressor lncRNA that regulates the expression of CYLD and SOX4, inducing a reduction in proliferation and invasion of melanoma cells.

Other lncRNAs influence cell proliferation and motility through their ability to act as a molecular “guide” by recruiting specific chromatin-modifying enzymes, thereby modifying the gene expression [69]. Indeed, the lncRNAs FOXC2-AS1 [54] and FALEC [55] modulate cell proliferation by recruiting EZH2 in the nucleus and induces the epigenetic silencing of p15 and p21, respectively. The expression of EZH2 in melanoma cells is reported to be also regulated by other two different tumor suppressor lncRNAs, i.e., LINC-PINT [56] and by GAS5 [57], but using different strategies. In detail, LINC-PINT, by directly recruiting EZH2, affects in vivo tumor growth in a xenograft model [56]; GAS5 inhibits cell apoptosis and oxidative stress by recruiting E2F4 that reduces EZH2 expression [57].

Recent findings revealed that the same lncRNA possess characteristics that acts by using multiple strategies of action. In this context, the lncRNA MIR205HG promotes cell proliferation and motility by mediating the stabilization of JMJD2C protein and by increasing the expression of ALKBH5 at transcriptional level [58]. In similar manner, the lncRNA SLNCR is upregulated in melanoma tissues and cell lines, silencing the expression of the tumor suppressor p21 through the recruitment of AR/EGR1 [59]. Additionally, the SLNCR1 isoform enhances proliferation and motility by increasing the expression of SOX5 [60] and by silencing SPRY2 [61].

Coherently with its role in several cancers, another important melanoma-associated lncRNA is SPRY4-IT1 [70]. High levels of SPRY4-IT1 in the plasma of melanoma patients are closely associated with tumor stage and a lower overall survival [71]. In melanoma cells, silencing of SPRY4-IT1 significantly reduces cell proliferation and migration and modulates the EMT markers [63,64,65].

Finally, emerging studies showed that HOTAIR is an important lncRNA increased in the plasma and tissues of several human cancer patients [72,73,74]. In the plasma of melanoma patients, its expression result is increased [75], while in melanoma cells its silencing influences the proliferation and apoptosis through the NF-ϰB pathway [66] and affects cell motility through the degradation of gelatin matrix [67]. Overall, these data suggest the potential role of HOTAIR as novel therapeutic agent in melanoma.

Another mechanism increasing the aggressiveness of melanoma cells by lncRNAs is due to their capability to regulate the expression of EMT-related genes and the expression of MMPs involved in distant metastases insurgence (Table 2).

For instance, the lncRNAs SLNCR1, previously mentioned, is also able to regulate both in vitro and in vivo the EMT markers by regulating the expression of SOX5 [60].

The upregulation of lncRNA CASC15 [76] and Gm33149 [77] in melanoma cells increase cell proliferation and migration through the activation of the important Wnt/beta-Catenin pathway, suggesting their role as oncogenes. On the contrary, a lncRNA characterized by a tumor suppressor activity is MEG3 since its downregulation activates Wnt pathway through the regulation of miR-21/E-Cadherin axis leading to a mesenchymal phenotype [78,79].

The upregulation of mesenchymal markers is mediated by LINC00662 [80], HOXA11-AS [82], SPRY4-IT1 [65], AFAP1-AS1 [83], and NEAT1 [46], which act as ceRNAs by competitively binding miR-107, miR-152-3p, miR-22-3p, miR-653-5p, and miR-200b-3p, respectively. Furthermore, miR-126 modulates the expression of LINC00888 and its target CRK, influencing proliferation, motility, and EMT marker expression [81].

Finally, regarding the regulation of MMPs, the literature indicates that the well-known oncogenic lncRNA, MALAT1, is upregulated in melanoma where, by sponging the miR-22, increases the expression of MMP14 and SNAIL [84]. MMP2 is also upregulated following the downregulation of lncRNA GAS5 [85].

### 2.2. Role of lncRNAs in Drug Resistance

Recent advancements in the understanding of lncRNAs function, highlighted that the aberrant expression of these molecules is also implicated in the lower antitumoral efficacy of chemotherapy and of target therapy (e.g., MAPK inhibitors and immunotherapy) in melanoma. One of reasons for this is attributable to lncRNAs ability to regulate the expression of genes targeted by these pharmacological treatments (Table 3).

For instance, resistance to dacarbazine-based chemotherapy is mediated by the overexpression of POU3F3 [86] that impairs the binding between MGMT and miR-650, an miRNA involved in the progression of several human cancers [96,97].

In melanoma cells, the overexpression of lncRNA H19 [87] and of NEAT1 [88] leads to the acquired resistance to cisplatin through the direct targeting to the miR-18b/IGF1 and miR-519c-3p/MeCP2 axes, respectively. Additionally, the silencing of the previously mentioned lncRNA, TUG1, impairs in vivo tumor growth and improved the chemosensitivity either to cisplatin or to fluorouracil (5FU) [49].

On the other hand, several lncRNAs are correlated to the MAPK inhibitor resistance and consequently to a poor prognosis in melanoma patients. The high levels of lncRNA LENOX [90] and of lncRNA U731166 [91] in melanoma tissues and cell lines are correlated with the acquired resistance to the vemurafenib. Also, the lncRNA MEG3 has been shown to be involved in the vemurafenib resistance via the activation of the HGF/c-MET pathway [92]. Furthermore, a low expression of the lncRNA TSLNC8 tumor suppressor is reported to be implicated in the reactivation of the MAPK pathway following PLX4720 treatment [93].

All these data suggest that melanoma patients could benefit from the combined treatment of MAPK inhibitors and lncRNAs delivery to overcome acquired clinical resistance.

Additionally, the aberrant expression of lncRNAs is closely related to immunotherapy response and to resistance to the immune checkpoint inhibitors [98]. Interestingly, a recent in silico study conducted on The Cancer Genome Atlas (TCGA) reveals 4-lncRNA signature correlated to the immunotherapy response in 71 melanoma patients [99]; likewise, 15-lncRNA signature was found in 92 melanoma patients associated with a anti-PD-1 monotherapy response [100].

In this context, increased expression level of LIMIT in melanoma patients is associated to an increased anti-PD-1 therapy efficacy, suggesting its potential utility in the clinic to enhance immunotherapy response [94].

Other results suggest that the higher expression of NEAT1 occurs in melanoma patients showing a complete response to anti-PD-1/PD-L1 immunotherapy [89].

The relationship between lncRNAs and the PD-1/PD-L1 pathway in melanoma is also confirmed in the study by Ding et al. The authors showed that the high level of LINC01214 promotes immunotherapy resistance through the regulation of the miR-4492/PPP1R11 axis, which induces the dysfunction of CD8+ T cells [95].

## 3. Application of lncRNAs as a Therapeutic Tool

### 3.1. Targeting lncRNAs Using RNA-Based Strategies

Consistent with the increasing evidence suggesting that lncRNAs play a pivotal role in the modulation of an aggressive phenotype in the acquisition of drug resistance and in the immunotherapy escape of melanoma cells, several strategies were developed to restore the expression of lncRNAs in melanoma patients.

Among the possible RNA-based strategies employed to silence the expression of oncogenic lncRNAs, there are small interfering RNAs (siRNAs) and antisense oligonucleotides (ASOs) [101].

Mechanistically, siRNAs are double-stranded RNA molecules composed of 20–25 base pairs that act directly in the nucleus that silence the lncRNA expression [102].

ASOs are single-stranded RNA molecules, 12–30 oligonucleotide long, that act both in cytoplasm and at the nuclear level, inhibiting the lncRNA function [103]. It is important to underline that it is possible to increase the stability of siRNA or ASO, increase their specificity and cellular uptake, and reduce their potential toxicity, by adding specific chemical modifications to their sequences [101]. Actually, these strategies are currently approved in several human diseases [103]. In melanoma, ASO strategy is used at the preclinical level to inhibit the expression of oncogenic MALAT1 [104]. Concerning consequences, MALAT1 ASO-based inhibition impacts negatively on the activation of the MAPK signaling pathway, impairing cell growth [104]. Notably, the silencing of MALAT1 is also obtained by the generation of small-molecule compounds that target this lncRNA, inducing its downregulation and suggesting the potential success of this strategy in melanoma [105]. Concerning the use of siRNA in melanoma, Xu and colleagues reported the treatment with a specific siRNA complementary to the ANRIL lncRNA reduces tumor growth in vivo [106].

LncRNA targeting could also be obtained by genome editing [101]. In particular, the CRISPR-Cas9 technique is a promising therapeutic tool that allows for reactivating or silencing the lncRNA expression. The aim of this technology is to permanently correct the alteration in specific regions of DNA sequences in cancer cells [101]. Indeed, recent studies showed that the CRISPR-Cas9 system is a versatile and accurate methodology to regulate the lncRNA expression in cancer therapy [107]. Recent studies conducted in a plethora of cancers showed the success of CRISPR-Cas9 technology to effectively knock out the expression of HOTAIR, NEAT1, XIST, TUG1, and UCA1 and to effectively upregulate the expression of MEG3 [107].

### 3.2. Targeting of lncRNAs Using Phytochemicals

Among innovative approaches directed toward the identification of complementary strategies for the treatment of melanoma, the use of plant-derived bioactive compounds arouses great interest [108]. Among these natural compounds, phytochemicals include a wide class of natural compounds derived from fruits, vegetables, tea, and roots with various chemical structure and functions [109].

For their biological properties, these natural compounds and their derivates exert many beneficial effects on human health and disease prevention, and exert anticancer properties, generally without any relevant side effects [110,111,112,113,114].

In particular, the antineoplastic effects of these natural products are due to their ability to target one or often multiple oncogenic signaling pathways involved in the progression of several types of human cancer, including melanoma [111,112,113,115,116].

For instance, phytochemicals (e.g., curcumin, resveratrol, and epigallocatechin-3 gallate) can inhibit, although to a different extent, the MAPK and WNT/β-catenin signaling pathways, thus reducing melanoma tumor growth and progression [117]. Additionally, quercetin, resveratrol, and sulforaphane are mostly involved in the downregulation of factors acting in the PI3K/AKT/mTOR pathway, which reduces melanoma metastatic potential [118].

Regarding inflammation that contributes to melanoma progression, it has been shown that quercetin exhibits anti-inflammatory and apoptotic effects in melanoma cells [119,120,121]. Epigallocatechin-3 gallate (EGCG), curcumin, and resveratrol inhibit VEGF expression level, reducing tumor vascularization [122].

In addition, phytochemicals show immunomodulation activity on various populations of immune cells involved in the mechanisms by which melanoma cells can escape the immune response [123].

Recent literature shows a link between the phytochemical administration and the expression of lncRNAs in human tissues [124]. In this context, a plethora of studies clearly demonstrated that phytochemicals exert anticancer effects that modulate the tumor suppressor or oncogenic lncRNA expression, mainly through epigenetic changes [125,126].

The main findings concerning the influences of phytochemical on melanoma-related lncRNAs are reported in Table 4.

Most of the studies published concern the effects of curcumin on lncRNAs expression in other cancers; nevertheless, in general, these lncRNAs targeted by phytochemicals play also an important role in melanoma.

For example, the administration of curcumin also exerts an antitumor effect in several types of cancer by inhibiting the expression of HOTAIR, an important oncogenic lncRNA [127,129]. As mentioned earlier, HOTAIR overexpression contributes to the metastatic progression of melanoma [66,67,75].

Abolfathi and colleagues revealed that curcumin reduces HOTAIR in a glioblastoma cell line, but also increased expression of lncRNA MEG3, a known tumor suppressor in melanoma [128].

Curcumin has also been linked to a higher level of MEG3 in ovarian cancer [137] and in non-small cell lung carcinoma (NSCLC) [135], which increases the sensitivity to cisplatin and reduces tumor growth, respectively. Through epigenetic change, curcumin also increased MEG3 expression in hepatocellular cancer cells [124].

Other studies on curcumin have shown that in glioma [131], breast [130], gastric [132], and hepatocellular [136] cancers, this natural compound reduces the aggressiveness of cancer cells by inhibiting the expression of H19, an oncogenic lncRNA also involved in the cisplatin response from melanoma cells [87].

In pancreatic cancer, curcumin administration suppressed the lncRNA PVT1 expression, increasing the sensitivity to gemcitabine [134]. The overexpression of this lncRNA in melanoma increases cell proliferation by sponging the miR-26b [62].

Resveratrol is another natural compound that in several cancers alters the expression of lncRNAs [147]. Some of these lncRNAs targets of resveratrol are also known to be correlated to the aggressiveness of melanoma (Table 4). For instance, in gastric cancer, Li and colleagues showed that resveratrol treatment reduces proliferation, migration, and endoplasmic reticulum stress by reducing the expression of H19 [138]. Interestingly, an inhibition of the Wnt/β-catenin signal pathway occurred in colorectal cancer and in multiple myeloma cells following resveratrol treatment via the silencing of MALAT1 [139] and of NEAT1 [141], respectively. Also, Yang and colleagues demonstrated that the silencing of MALAT1 by resveratrol inhibits the proliferation and motility in gastric cancer by the modulation of the miR-383-5p/DDIT4 axis [140].

Among the flavonoids, genistein, EGCG, and quercetin influence the expression of several lncRNAs implicated in the pathogenesis of melanoma.

Genistein is a isoflavone that exerts antitumor activity in prostate [142] and in breast [143] cancer cells by reducing the expression of the oncogenic lncRNA HOTAIR.

EGCG reduces the expression of NEAT1 in lung cancer by increasing the sensitivity to cisplatin [144].

Quercetin inhibits the expression of MALAT1 in prostate [145] and in breast [146] cancers.

Overall, these studies suggest that these natural compounds could also exert their anticancer effects in melanoma by restoring the correct expression of these lncRNAs.

## 4. Nanodelivery Strategies for RNA-Based Therapy Targeting lncRNAs in Melanoma Cells

It is clear that for the success of therapies based on nucleic acid and phytochemicals targeting a specific lncRNA, is crucial to obtain a specific delivery system directed toward melanoma cells. In this frame, nanotechnology has provided several strategies to guarantee with high affinity and specificity the efficient transfer of nucleic acid targeting lncRNAs into cancer cells [148]. This is important to mitigate potential side effects, to avoid the instability in blood, and to increase cell uptake and safety. Furthermore, these delivery systems improve the bioavailability, stability, and targeted delivery of RNA-based drugs, thereby increasing their therapeutic efficacy [148,149,150].

Lentiviral vectors (LVs) represent the first delivery system designed to prevent the degradation of lncRNAs-based drugs and, consequently, guarantee their efficient delivery [148]. For instance, overexpression of lncRNA GAS5 via lentiviral vectors has been shown to inhibit migration and invasion in melanoma cells by downregulating MMP2 expression and activity [85]. Even if LVs are characterized by a highly efficient delivery into cancer cells and guaranteed long-expression time, LVs are also characterized by potential toxicity. This includes risks such as insertional mutagenesis, where integration into the host genome can disrupt normal gene functions, leading to oncogene activation or tumor suppressor gene inactivation, which may increase cancer risks [151].

Recent advances in nanomedicine have led to the development of specific nanoparticles (NPs) characterized by different biological properties, size, and different modified membranes to optimize the delivery of the drug into the tumor sites.

The most common NPs developed are composed of ionizable lipids, polyethylene glycol (PEG) and cholesterol, which confer high stability [148]. Other nanocarrier formulations include NPs composed of polymers, such as polyethyleneimine, ranging from 50 to 200 nm in diameter, where RNA can be loaded through electrostatic interactions [148]. 

Other nanocarrier formulations currently developed involve the use of magnetic NPs composed by porous iron oxide. However, their use is hindered by their low solubility and potential toxicity [152]. Studies have shown that ultrasmall iron oxide NPs can induce significant toxicity, including inflammation and decreased cell viability [153].

A more recent and promising approach to the acid nucleic delivery system is represented by natural carriers, i.e., extracellular vesicles (EVs). EVs are small carriers (ranging from 30 to 200 nm) used by cancer cells to facilitate their cell-to-cell communication. Indeed, they are important mediators of tumor development, progression, metastasis insurgence, and drug resistance [154,155]. Cancer therapies based on EVs offer several advantages such as the high tolerability and low immunogenicity [156,157]. Challenges include high cost, low production efficiency during their isolation, and aggregation during storage (59).

Regarding the restoration of lncRNAs in cancer cells, an innovative method is represented by using the encapsulating system CRISPR/Cas9 inside them to modify the expression of oncogenic genes into targeted cells exploiting the EVs [158]. This strategy has shown potential in preclinical studies, demonstrating effective gene editing with reduced off-target effects [159].

Additionally, another potential strategy to target oncogenic lncRNAs is represented by the use of the oncolytic adenovirus as a delivery system. This virus system can be used as vector capable to deliver designed lncRNA interference into cancer cells.

One of the major advantages of this method is represented by the capability of the adenovirus expressing the lncRNA intereference to infect only cancer cells where it can accumulate in high copy number, without infecting healthy cells. Aside from the non-pathogenicity of this viral system, the oncolytic virus results are stable and free from immune response, suggesting its potential clinical application. Indeed, recombinant adenoviruses expressing specific lncRNA interference were already designed in several cancers, e.g., hepatocellular carcinoma [160] and breast cancer [161]; however, the antitumor efficacy of the oncolytitc virus against lncRNA in melanoma is missing.

While various delivery systems such as LVs, NPs, and EVs have been developed to transport lncRNAs into melanoma cells, each system presents challenges. Ongoing research aims to optimize these delivery methods to enhance specificity, reduce toxicity, and improve therapeutic outcomes in melanoma treatment.

## 5. Conclusions

By modulating several signaling pathways, LncRNAs are highly conserved nucleic acids that are crucial regulators of cancer hallmarks. Consequently, restoring the correct expression of lncRNAs in cancer tissues could have important benefits against cancer progression.

Broadly viewed, our literature analysis revealed that HOTAIR, H19, and MALAT1 are the main oncogenic lncRNAs studied, and correlated to the aggressiveness of melanoma phenotype and in the acquisition of drug resistance.

Indeed, in a wide spectrum of human cancers, it is reported that the expression of these lncRNAs can be modulated by the administration of several phytochemicals. Phytochemicals can be considered coadjutants in cancer therapy for their low toxicity, low cost, and high compliance when used as dietary supplements. In addition, the integration of phytotherapy with immune and target therapies might enhance the efficacy of conventional therapies. In particular, the main phytochemicals involved in the lncRNA modulation are curcumin and resveratrol that, restoring the expression of lncRNAs, e.g., H19, HOTAIR, and MALAT1, lead to the inhibition of cellular proliferation and motility in several cancer types: breast, glioma, gastric, renal, and colorectal.

Although phytotherapy needs further investigation and research, this approach shows promise in improving treatment outcomes, especially in chemo-resistance to traditional therapy.

Importantly, to the best of our knowledge, the role of phytochemicals on lncRNA expression in melanoma is not unveiled. Thus, other studies should also be performed to uncover the lncRNAs/phytochemicals relationship in melanoma patients.

Indeed, despite the current knowledge regarding the potential synergistic effects of lncRNAs and phytochemicals in various cancers, preclinical and clinical investigations in melanoma are lacking.

Further, novel research could be performed to explore the combined effects of phytochemicals on lncRNAs with conventional and modern anticancer treatments to increase their sensitivity and hinder the insurgence of drug resistance.

In light of this, significant advantages will be provided by the progression in nanotechnology, allowing the effective delivery of these molecules into the tumor site, improving their availability, and reducing the risk of side effects through the formulation of specific nanocarriers.

## Figures and Tables

**Table 1:** List of lncRNAs aberrantly expressed in melanoma and their effects on cell proliferation and invasiveness.

LncRNA	Expression	Function	Mechanism of Action	Targets	Ref., Year
BANCR	↑ Tissues/cell lines	Oncogene	ceRNA	miR-204/NOTCH-2	[40], 2017
SNHG5	↑ Tissues/cell lines	Oncogene	ceRNA	miR-26a-5p/TRPC3	[41], 2018
ATB	↑ Tissues/cell lines	Oncogene	ceRNA	miR-590-5p/YAP1	[42], 2018
LINC00662	↑ Tissues/cell lines	Oncogene	ceRNA	miR-890/ELK3	[43], 2020
NEAT1	↑ Tissues/cell lines	Oncogene	ceRNA	miR-495-3p/E2F3	[44], 2019
				miR-23a-3p/KLF3	[45], 2019
				miR-200-3p/SMAD2	[46], 2020
FOXD3-AS1	↑ Tissues/cell lines	Oncogene	ceRNA	miR-127-3p/FJX1	[47], 2020
				miR-325/MAP3K2	[48], 2019
TUG1	↑ Tissues/cell lines	Oncogene	ceRNA	miR-129-5p/AEG-1	[49], 2018
				miR-29c-3p/RGS1	[50], 2019
				miR-145-5p/SOX2	[51], 2022
MEG3	↓ Tissues/cell lines	T. Suppressor	ceRNA	miR-499-5p/CYLD	[52], 2018
				miR-208/SOX4	[53], 2023
FOXC2-AS1	↑ Tissues/cell lines	Oncogene	guide	EZH2/p15	[54], 2020
FALEC	↑ Tissues/cell lines	Oncogene	guide	EZH2/p21	[55], 2017
LINC-PINT	↓ Tissues/cell lines	T. Suppressor	guide	EZH2	[56], 2019
GAS5	↓ Tissues/cell lines	T. Suppressor	guide	E2F4/EZH2	[57], 2020
MIR205HG	↑ Cell lines	Oncogene	guide	JMJD2C ALKBH5	[58], 2024
SLNCR	↑ Tissues/cell lines	Oncogene	guide	AR/EGR1/p21	[59], 2019
SLNCR1	↑ Tissues/cell lines	Oncogene	guide	SOX5	[60], 2024
				DNMT1/SPRY2	[61], 2024
PVT1	↑ Tissues/cell lines	Oncogene	ceRNA	miR-26b	[62], 2018
SPRY4-IT1	↑ Cell lines	Oncogene	-	-	[63], 2011
	↑ Cell lines		-	Lipin 2	[64], 2014
	↑ Cell lines		-	miR-22-3p	[65], 2019
HOTAIR	↑ Tissues/cell lines	Oncogene	-	NF-ϰB pathway	[66], 2020
				Gelatinase activity	[67], 2013

**ceRNA**: competing endogenous RNA; **EMT**: epithelial to mesenchymal transition, **T. Suppressor**: tumor suppressor; **↑**: increased; **↓**: decreased.

**Table 2:** List of lncRNAs aberrantly expressed in melanoma and their effects on EMT.

LncRNA	Expression	Function	Mechanism of Action	Targets	Ref., Year
SLNCR1	↑ Tissues/cell lines	Oncogene	guide	SOX5	[60], 2024
CASC15	↑ Tissues/cell lines	Oncogene	-	Wnt/β-Catenin pathway	[76], 2020
Gm33149	↑ Cell lines/ exosome secreted by CSCs	Oncogene	ceRNA	miR-5623-3p/Wnt pathway	[77], 2024
MEG3	↓ Tissues/cell lines	T. Suppressor	ceRNA	miR-21/E- cadherin	[78], 2020
	↓ Cell lines		-	Wnt/β-Catenin pathway	[79], 2018
LINC00662	↑ Tissues/cell lines	Oncogene	ceRNA	miR-107/POU3F2	[80], 2024
LINC00888	↑ Tissues/cell lines	Oncogene	-	CRK	[81], 2018
HOXA11-AS	↑ Tissues/cell lines	Oncogene	ceRNA	miR-152-3p/ITGA9	[82], 2021
SPRY4-IT1	↑ Cell lines	Oncogene	-	miR-22-3p	[65], 2018
AFAP1-AS1	↑ Cell lines	Oncogene	ceRNA	miR-653-5p/RAI14	[83], 2020
MALAT1	↑ Tissues/cell lines	Oncogene	ceRNA	miR-22/MMP14/Snail	[84], 2016
GAS5	↓ Cell lines	T. Suppressor	-	MMP2	[85], 2016
NEAT1	↑ Tissues/cell lines	Oncogene	ceRNA	miR-200b-3p/SMAD2	[46], 2020

**ceRNA**: competing endogenous RNA; **CSC**: cancer stem cells; **EMT**: epithelial mesenchymal transition; **T. Suppressor**: tumor suppressor; **↑**: increased; **↓**: decreased.

**Table 3:** List of lncRNAs aberrantly expressed in melanoma and their effects on treatment response.

LncRNA	Expression	Function	Mechanism of Action	Targets	Acquisition of Resistance to	Ref., Year
POU3F3	↑ Tissues/cell lines	Oncogene	ceRNA	miR-650/MGMT	Dacarbazine	[86], 2021
TUG1	↑ Tissues/cell lines	Oncogene	ceRNA	miR-129-5p/AEG1	Cisplatin and 5-FU treatment	[49], 2018
H19	↑ Tissues/cell lines	Oncogene	ceRNA	miR-18b/IGF1	Cisplatin	[87], 2020
NEAT1	↑ Tissues/cell lines	Oncogene	ceRNA	miR-519c-3p/MeCP2	Cisplatin	[88], 2023
	↑ Tissues/cell lines	Oncogene	-	-	anti PD-1 immunotherapy	[89], 2023
LENOX	↑ Tissues/cell lines/PDX tumors	Oncogene	Guide	RAP2C	Vemurafenib	[90], 2022
U731166	↑ Tissues/cell lines	Oncogene	-	-	Vemurafenib	[91], 2022
MEG3	↓ Tissues/cell lines	T. Suppressor	-	HGF/c-MET pathway	Vemurafenib	[92], 2024
TSLNC8	↓ Tissues/cell lines	T. Suppressor	Guide	PP1α	PLX4720	[93], 2021
LIMIT	↓ Tissues/cell lines	T. Suppressor	-	MHC-1	anti PD-1 immunotherapy	[94], 2021
LINC01214	↑ Tissues/plasma/cell lines	Oncogene	ceRNA	miR-4492/PPP1R11	anti PD-1 immunotherapy	[95], 2024

**ceRNA**: competing endogenous RNA; **FU**: Fluorouracil, **PDX**: patient-derived xenograft; **T. Suppressor**: tumor suppressor; **↑**: increased; **↓**: decreased.

**Table 4:** List of lncRNAs altered in melanoma and targets of phytochemicals in other types of human cancer.

Phytochemical	Source	lncRNA Target	Cancer	Effects	Ref., Year
Curcumin	Turmeric	↓ HOTAIR	Renal	↓ Migration	[127], 2014
			Glioblastoma	Gene expression change	[128], 2023
			AML	↓ Drug resistance	[129], 2021
		↓ H19	Breast	↓ EMT	[130], 2021
			Glioma	↑ Expression of VDR ↓ Tumor volume	[131], 2020
			Gastric	↓ Proliferation	[132], 2016
			Hepatocellular	↓ Proliferation	[133], 2021
		↓ PVT1	Pancreatic	↑ Drug sensitivity	[134], 2017
		↑ MEG3	NSCLC	↓ Tumor growth	[135], 2021
			Hepatocellular	Epigenetic change	[136], 2015
			Ovarian	↓ Drug resistance	[137], 2017
			Glioblastoma	Gene expression change	[128], 2023
Resveratrol	Peanuts, grapes, red wine	↓ H19	Gastric	↓ Proliferation, migration, ER stress	[138], 2022
		↓ MALAT1	Colorectal	↓ Migration	[139], 2013
			Gastric	↓ Proliferation, migration,	[140], 2022
		↓ NEAT1	Multiple myeloma	↓ Proliferation, motility	[141], 2018
Genistein	Fruits, vegetable, tea	↓ HOTAIR	Prostate	↓ Proliferation	[142], 2013
			Breast	↑ Apoptosis	[143], 2015
EGCG	Fruits, vegetable, tea	↓ NEAT1	NSCLC	↑ cisplatin sensitivity	[144], 2016
Quercetin	Fruits, vegetable, tea	↓ MALAT1	Prostate	↓ Proliferation, motility, EMT	[145], 2020
			Breast	↓ Proliferation, motility	[146], 2022

**AML**: acute myeloid leukemia; **EGCG**: epigallocatechin-3-gallate; **EMT**: epithelial mesenchymal transition; **ER**: endoplasmic reticulum; **NSCLC**: non-small cell lung carcinoma; **VDR**: vitamin D receptor; **↑**: increased; **↓**: decreased.

## Abbreviations

AML	acute myeloid leukemia
ASO	antisense oligonucleotides
CAF	tumor-associated fibroblast
ceRNA	competing endogenous RNA
CSC	cancer stem cells
CTLA-4	Cytotoxic T-lymphocyte associated protein 4
EGCG	Epigallocatechin gallate
EMT	epithelial–mesenchymal transition
ER	endoplasmic reticulum
EV	extracellular vesicle
FU	Fluorouracil
ICI	immune checkpoint inhibitor
IFN-γ	interferon gamma
lncRNA	long non-coding RNA
LAG-3	Lymphocyte activation gene 3 protein
LV	lentiviral vector
MAPK	Mitogen-activated protein kinase
MHC-I	Major histocompatibility complex I
miRNA	microRNA
NPs	nanoparticles
NSCLC	non-small cell lung carcinoma
PD-1	Programmed cell death protein 1
PD-L1	Programmed death-ligand 1
PEG	polyethylene glycol
PDX	patient-derived xenograft
VDR	vitamin D receptor
siRNAs	small interfering RNAs
TME	tumor microenvironment
T. Suppressor	tumor suppressor
